# Gel immersion endoscopy facilitates endoscopic ultrasound-guided jejunojejunostomy

**DOI:** 10.1055/a-2934-8475

**Published:** 2026-08-21

**Authors:** Hirotoshi Ishiwatari, Hiroki Sakamoto, Akihiro Ohba, Katsuhisa Ohgi, Teiichi Sugiura

**Affiliations:** 1 Division of Pancreatobiliary Medicine 38471 Shizuoka Cancer CenterSunto DistrictShizuokaJapan; 2 Division of Hepato-Biliary-Pancreatic Surgery 38471 Shizuoka Cancer CenterSunto DistrictShizuokaJapan

Endoscopic ultrasound-guided enteroenterostomy, including endoscopic ultrasound-guided jejunojejunostomy (EUS-JJ), is an emerging treatment for bowel obstruction, 1​ although its feasibility and safety have yet to be established. Gel immersion endoscopy using ViscoClear (Otsuka Pharmaceutical Factory, Japan), an electrolyte-free gel, secures the visual field and enables effective coagulation during endoscopic procedures. 2​ It may also help maintain intestinal distension during EUS-JJ. Herein, we report a case of EUS-JJ using gel-immersion endoscopy for adhesive bowel obstruction.

**Fig. 1:**
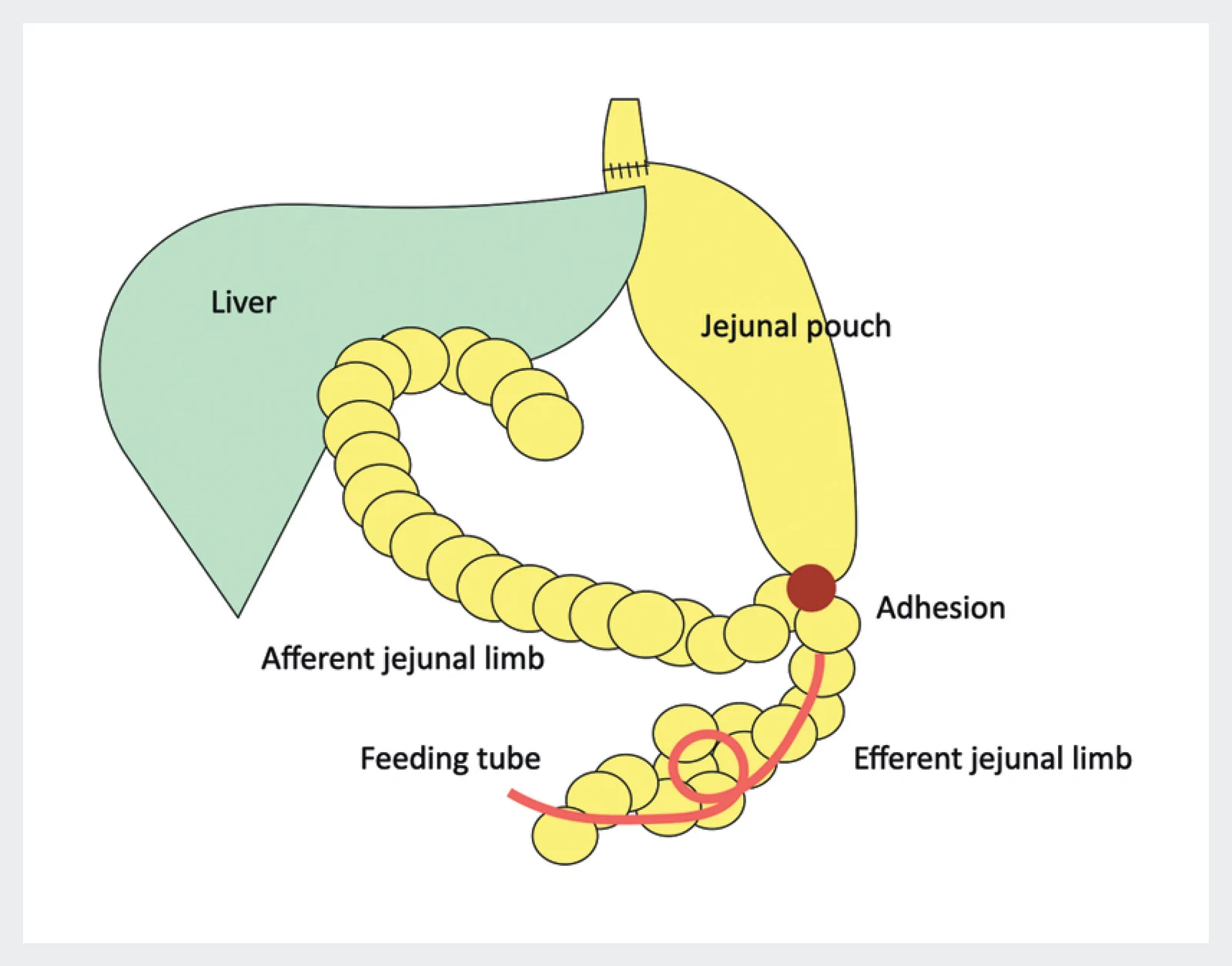
Schema of the surgically altered digestive anatomy in this case.

**Fig. 2:**
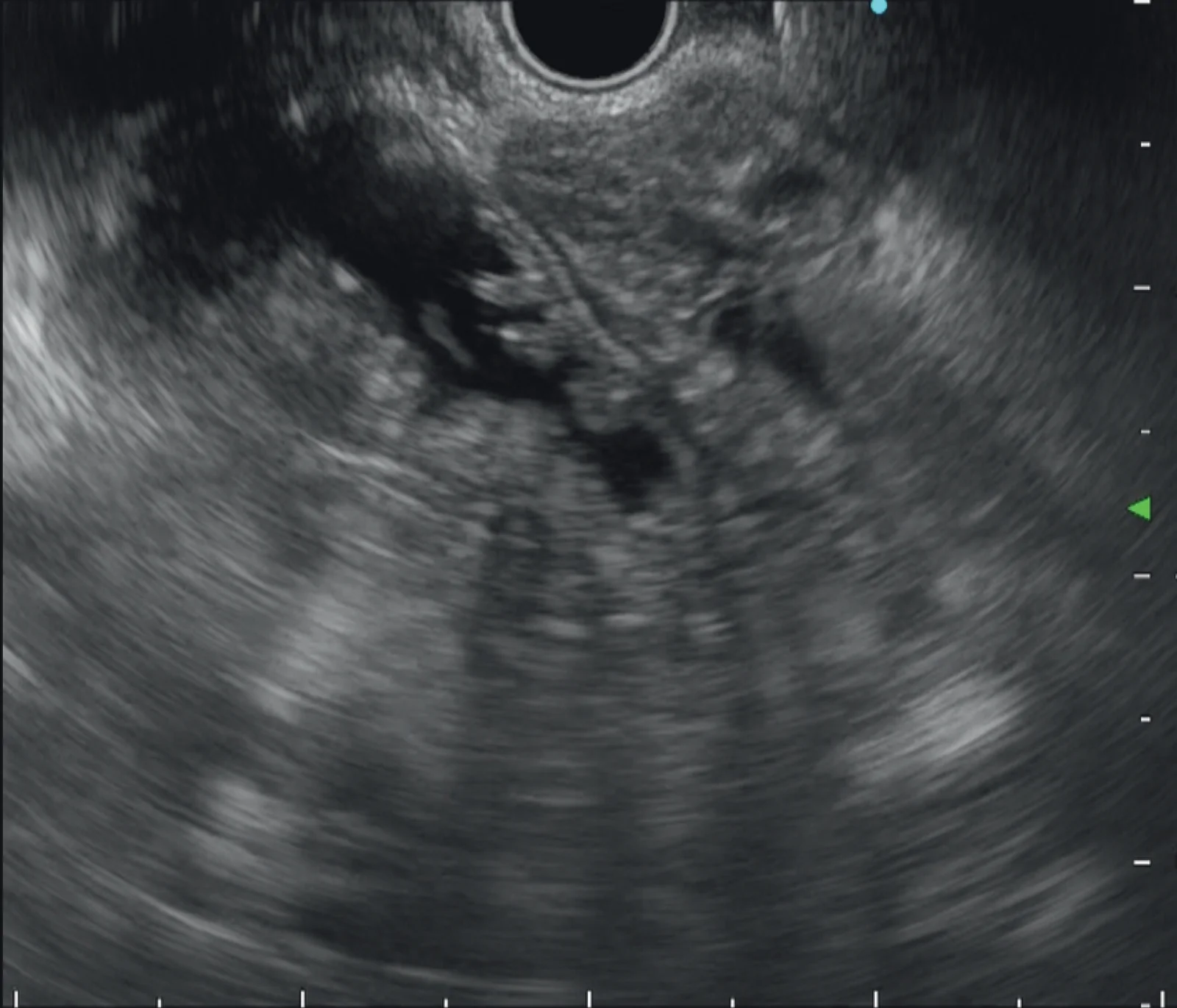
EUS findings from the jejunal pouch. Saline injection through the feeding tube dilates the jejunum but rapidly dissipates, failing to achieve adequate distension for the safe deployment of a LAMS.

**Fig. 3:**
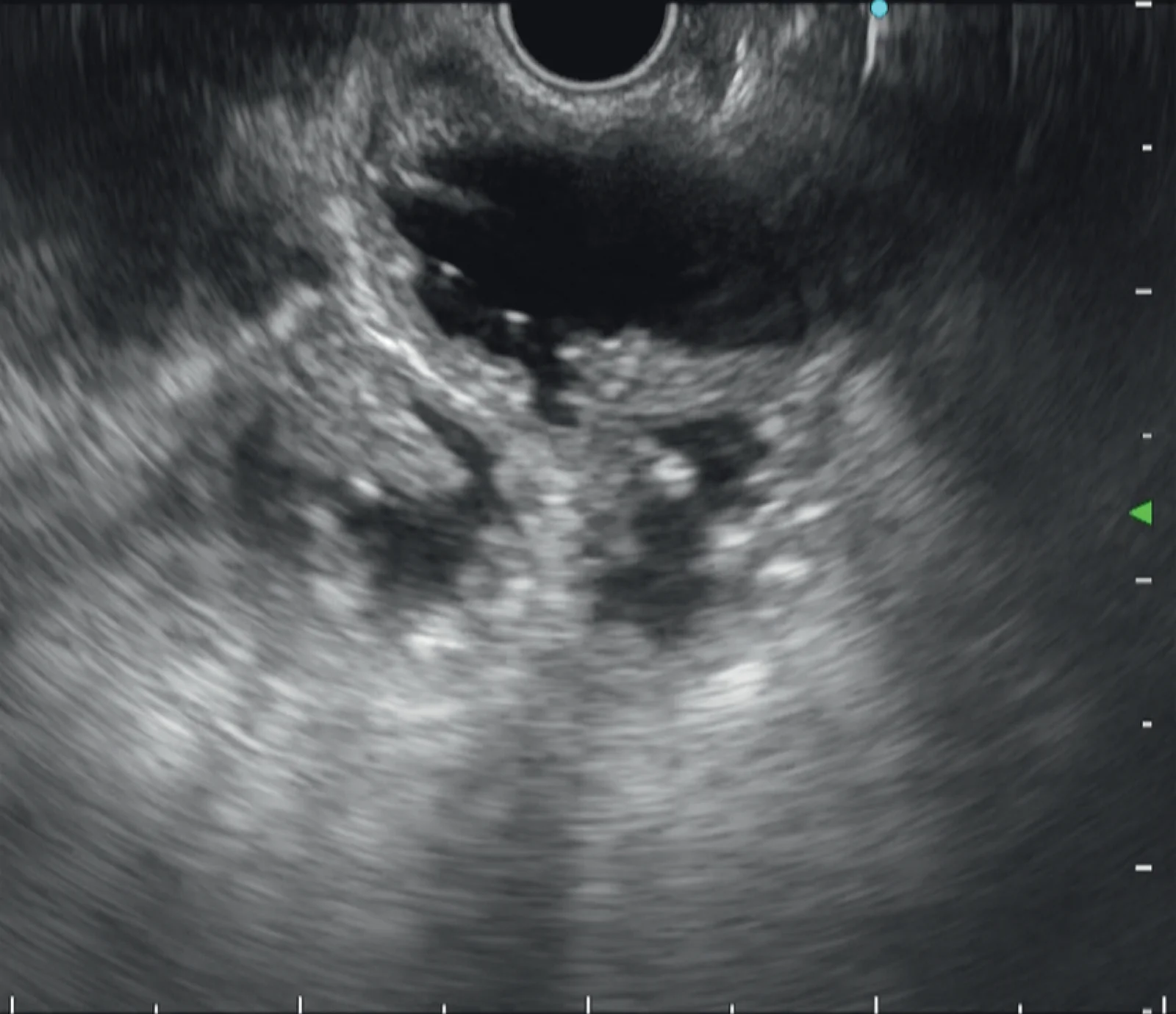
EUS findings from the jejunal pouch after ViscoClear injection. ViscoClear gel maintains adequate distension of the targeted jejunum, enabling safe stent deployment.

A 71-year-old man with a history of total gastrectomy for gastric cancer subsequently underwent pancreaticoduodenectomy for pancreatic cancer ( Video 1 ). On postoperative day (POD) 4, persistent vomiting led to aspiration pneumonia. Fluoroscopy showed contrast medium remaining in the jejunal pouch after esophagojejunostomy, and gastroscopy could not pass the obstructed anastomosis between the pouch and the jejunal limb ( Fig. 1 ). Surgical adhesiolysis was attempted on POD 23 but was unsuccessful because of dense adhesions. On POD 49, EUS-JJ was performed using a lumen-apposing metal stent (LAMS). EUS identified the jejunal limb adjacent to the pouch. Although saline was infused through a feeding tube placed in the efferent jejunal limb during the previous surgery, it drained rapidly despite antispasmodic administration, preventing adequate bowel distension for safe LAMS deployment ( Fig. 2 ). After injection of 200 mL of ViscoClear using a 20 mL syringe, the jejunal limb was sufficiently dilated. Despite a luminal diameter of less than 3 cm, a 15-mm LAMS (Hot-AXIOS; Boston Scientific) was inserted using cautery-assisted puncture and successfully deployed using a push deployment technique ( **Figs** 3 **and** 4 ). 3​ Contrast injection confirmed correct stent placement ( Fig. 5 ). Oral intake resumed the following day without adverse events. At the 6-month follow-up, the patient remained asymptomatic on a regular diet.

**Video 1** A clear, high-viscosity gel was used to maintain adequate distension of the target jejunum for the safe deployment of a lumen-apposing stent during EUS-guided jejunojejunostomy for adhesive bowel obstruction.


**Fig. 4:**
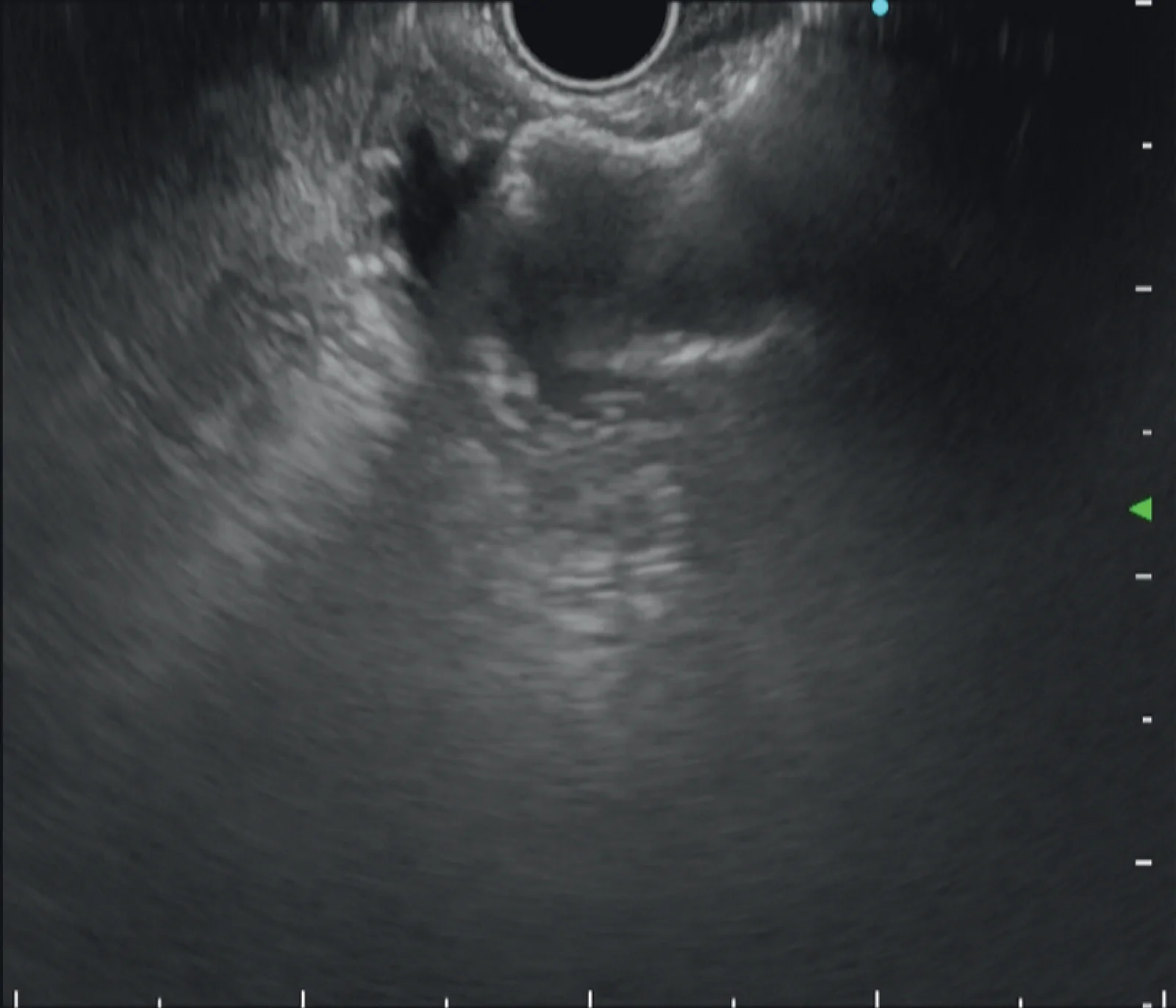
Deployment of a LAMS. EUS shows that the distal flange of the LAMS was successfully placed in the jejunum.

**Fig. 5:**
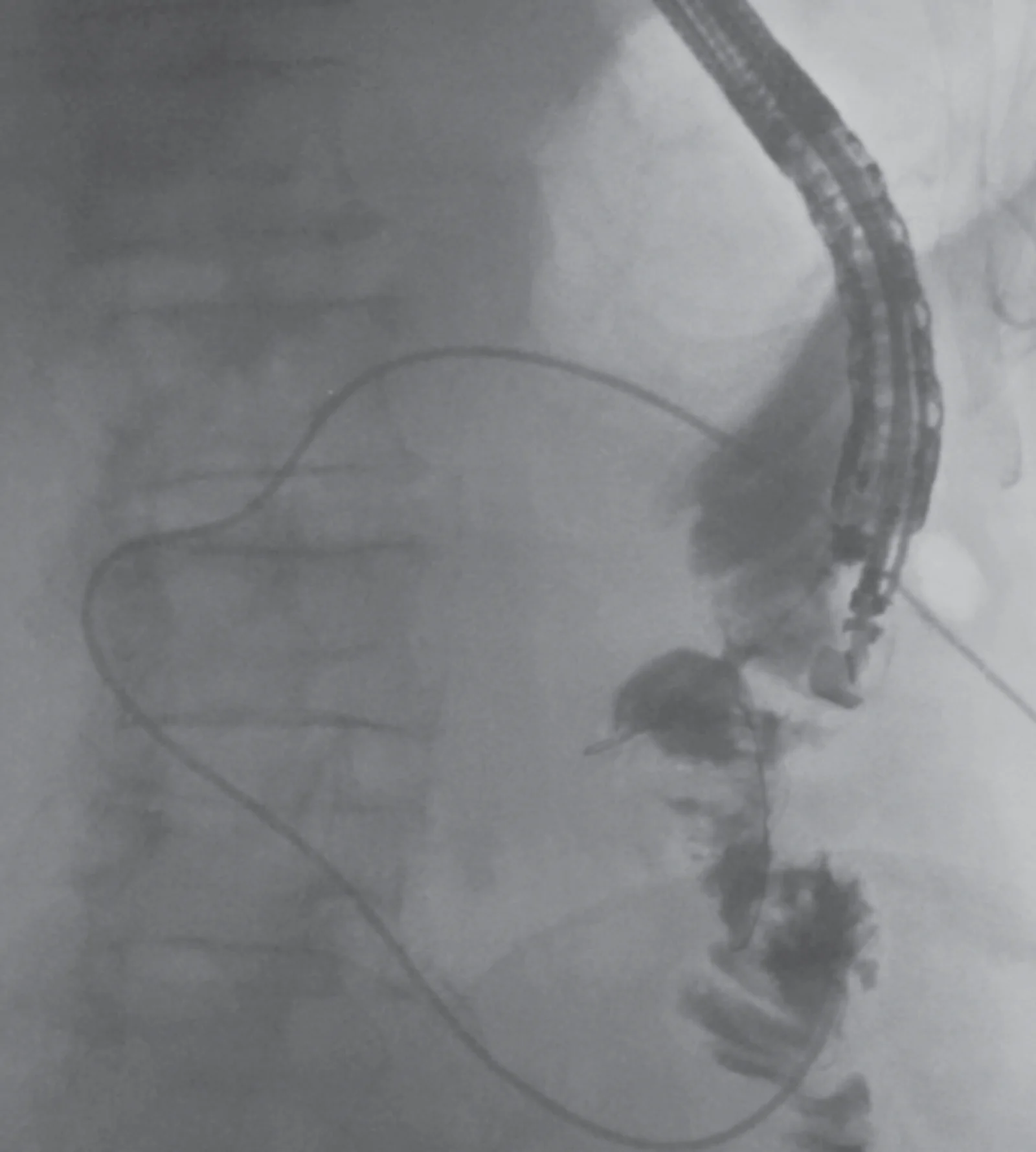
Contrast examination after LAMS deployment. Contrast injection through the feeding tube confirmed the successful LAMS deployment between the jejunal pouch and the jejunal limb.

This case suggests that an electrolyte-free gel may facilitate adequate intestinal distension during EUS-guided enteroenterostomy, particularly when saline infusion alone is insufficient to maintain the target lumen.

Endoscopy_UCTN_Code_TTT_1AS_2AK
